# Catch me if you can—emission patterns of human bodies in relation to postmortem changes

**DOI:** 10.1007/s00414-024-03194-3

**Published:** 2024-03-08

**Authors:** Alexandra Schieweck, Nicole Schulz, Jens Amendt, Christoph Birngruber, Franziska Holz

**Affiliations:** 1 Department of Material Analysis and Indoor Chemistry, Fraunhofer WKI, Riedenkamp 3, 38108 Braunschweig, Germany; 2 Institute of Legal Medicine, University Hospital Frankfurt, Goethe University, Kennedyallee 104, 60596 Frankfurt am Main, Germany

**Keywords:** Human corpses, Volatile organic compounds, Emissions, Olfactometry, GC-MS, Post-mortem interval (PMI)

## Abstract

The present study examines for the first time the emission patterns and olfactory signatures of 9 complete human corpses of different stages of decomposition. Air sampling was performed inside the body bags with solid sorbents and analysed by coupled gas chromatography-mass spectrometry after thermal desorption (TD-GC-MS). Furthermore, odour-related substances were detected by gas chromatography-olfactometry (GC-O). Sulfurous compounds (mainly dimethyl di- and trisulfide) were identified as most important to the odour perception. Around 350 individual organic substances were detected by TD-GC-MS, notably sulfurous and nitrogenous substances as well as branched alkanes, aldehydes, ketones, alcohols, carboxylic acids, carboxylic acid esters and ethers. A range of terpenes was detected for the first time in a characteristic emission pattern over all decomposition stages. Concentrations of the substances varied greatly, and no correlation between the emission patterns, the stage of decomposition and the cause of death could be found. While previous studies often analysed pig cadavers or only parts of human tissue, the present study shows the importance of analysing complete human corpses over a range of decomposition stages. Moreover, it is shown that using body bags as a kind of “emission test chamber” is a very promising approach, also because it is a realistic application considering the usual transport and store of a body before autopsy.

**Graphical abstract:**

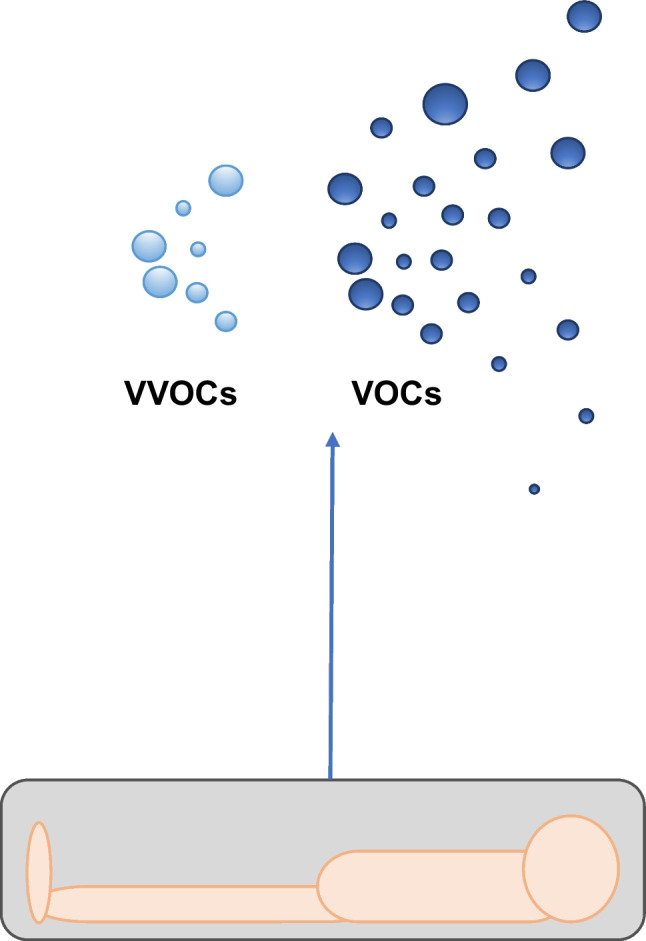

## Introduction

The identification of volatile organic compounds (VOCs), which are released during the decomposition of human bodies, is regarded as an important key element for detecting human remains open in the field or hidden under vegetation and debris, buried in the ground, or submerged in water [1–7]. Especially when it comes to train human remains detection (HRD) dogs, the knowledge of a unique VOC signature released by human bodies would be of major importance and help [8, 9]. But the analysis of the VOC composition could also be helpful to narrow down the post-mortem interval (PMI), especially for those cases in which the early post-mortem changes such as body temperature, rigor mortis and livor mortis can no longer be used [10, 11]. An overview of approaches for identifying the odour of death is given by Verheggen et al. [12].

Due to ethical and legal restrictions, pathological samples or tissues, which have been in close contact with human remains, are sometimes the only option to investigate corpse emissions [6, 13]. Instead, pig carcasses are commonly used as a human surrogate for investigating VOC emission profiles since the structure of their bodies has great similarities: relationship of fat to muscles, degree of hair coverage, weight and similar biochemical processes [6, 7, 14, 15]. However, it remains unclear whether the results can be transferred to the emission behaviour of a human corpse. As a matter of fact, several authors emphasized, that both the emission profile and the olfactive signature of decaying pig carcasses and decaying human bodies shows similarities and differences [16, 17]. Rosier et al. [13] investigated small samples of human and animal remains by headspace-GC-MS over a time period of six months under laboratory conditions in order to control environmental parameters and to standardize the test methodology. The authors assumed to identify eight substances as specific for human and pig samples out of over 400 volatile organics which had been detected in total. Those eight compounds comprised mainly esters as well as sulfurous and nitrogenous substances. In addition, five esters were selected by using principle component analysis (PCA) to separate human emissions from pig emissions. Cablk et al. [6] analysed animal tissue samples by headspace-GC-MS and compared the data with those published for human remains. Assuming that the same volatile organics that have been identified by SPME-GC-MS analysis are also responsible for the odour signature that can be detected by HRD dogs, chicken remains would have a greater similarity to human emissions than pig cadavers [6]. DeGreeff and Furton [18] investigated different materials for adsorbing odour-related volatiles and subsequent analysis by SPME-GC-MS. They identified a set of around 12 substances which they assumed to be unique for deceased human bodies as they showed differences to the odour of living persons and animals. The listed substances comprised higher alkanes (e.g. heptadecane, pentadecane), carboxylic acids, aldehydes, such as decanal and benzaldehyde, and aromatic hydrocarbons, such as styrene, xylene-isomers and phenol. However, all of these compounds are commonly detected in environmental air and might be released by different emission sources [18]. Statheropoulos and co-workers [3, 4] investigated the release of VOCs from a complete human body and listed few core substances which were different to those published before. Of course, the comparability and transferability of published data is always limited due to differing specimens, e.g. pig carcasses, animal tissues and human pathological samples, different experimental set-ups and varying analytical methods [5, 7, 15, 19]. During previous measurement campaigns, carcasses or test specimens have been stored within different kinds of enclosures. The air inside these enclosures was not always subjected to analysis, which makes a differentiation of background emissions from sample emissions impossible [1, 3]. Knobel et al. [20] sampled air over human and pig remains in the open Australian environment by allowing body surface air to diffuse into headspace vials. This approach has many risks and limitations as it cannot be guaranteed that the emission profile of the body will be catched without contamination with outdoor air. In addition, the sample volume is not defined. However, the study outlined visual differences between decaying human bodies and pig carcasses and, thus, concluded that pigs are not reliable analogous to humans in the early decomposition stages and in humid warm environments. Instead, based on VOC analysis, emission profiles of humans and pigs becoming more comparable the cooler the environment.

Hence, the metabolic reactions leading to specific VOC emissions are still largely unexplained [1, 13].

### Study objectives

Since the volatilome of human corpses is of great importance and, facing the circumstances that so far mostly pig carcasses or only small-sized pathological samples have been investigated, the present study aimed to examine the emission patterns and olfactory signatures of complete human corpses of different stages of decomposition. Thus, a broad profile of volatile organics, both very volatile organic compounds and volatile organic compounds (VVOC/VOC), released by the total body have been analysed. Furthermore, odour-related substances were detected for the first time by gas chromatography-olfactometry (GC-O).

## Experimental

### Human bodies

For emission measurements, human corpses, which have been taken to the Institute of Legal Medicine for autopsy, have been selected. Three different stages of decomposition were assigned: (1) fresh: no visible signs of decomposition, (2) initial signs of decomposition, and (3) advanced signs of decomposition. The bodies were naked or partially clothed depending on the circumstances of death. Until sampling, the corpses were stored in body bags in cooling chambers at a temperature of approximately 6 °C ± 1.5 °C. The number of bodies investigated in each decomposition stage, essential information regarding the cause and circumstances of death, post-mortem intervals (PMI) as well as applied air sampling are provided in Table 1. In this study, post-mortem intervals (PMI) are defined as the time between (anticipated) time of death and start of sampling. Air sampling was performed in an autopsy room prior the autopsy in order to avoid a dilution of the air inside the body bags by opening the zippers and treatments of the body, which could distort its emission characteristics. Thus, sampling tubes were inserted through a small gap of the closed zippers at the head of the body bags without touching the human bodies or the inner side of the bags.

**Table 1:** Overview of investigated bodies/body bags according to the decomposition stage with information about the cases and circumstances of dead, the post-mortem interval (PMI) as well as the applied air sampling

Stage	Body no	Cause of death	Circumstances of death	PMI (days)	Applied air sampling		
					VVOC	VOC	GC-O
1: Fresh	1	Pneumonia	Death in hospital	1	**−**	** + **	**−**
	2	Intoxication (medication)	Death in hospital	4	** + **	** + **	** + **
	3	Myocardial infarction	Death at workplace	5	** + **	** + **	** + **
2: Initial	4	Atypical hanging	Found dead at home	5–6	** + **	** + **	** + **
	5	Renal failure	Death in hospital	2	** + **	** + **	** + **
3: Advanced	6	Right heart failure	Found dead at home, putrefaction, single maggots	10–11	**−**	** + **	**−**
	7	Intoxication (carbon monoxide)	Found dead in a tent, partial skeletonization, abundant maggots	7–9	** + **	** + **	** + **
	8	Ketoacidosis (diabetes mellitus)	Found dead at home, partial skeletonization, few maggots	5	** + **	** + **	** + **
	9	Intoxication (drugs)	Found dead at home, partial skeletonization, lots of maggots	8	** + **	** + **	** + **
Autopsy room air (background values)					**−**	** + **	**−**

### Air sampling

The measurements were carried out in summer. The autopsy room was climatized to 22 °C and approx. 50% relative humidity. Sampling of body bag air was performed for analysing VOCs including odour assessment, as summarized in Table 1. Where possible, VVOC-analysis was additionally realized. In cases where this was not feasible, VVOC data presented in this paper were obtained by sampling via Tenax® TA, even though it is well-known that just a small range of VVOCs can be trapped by this adsorbent and that obtained results are not reliable [21]. In parallel, air sampling was performed in the autopsy room to reveal background values and undesired influences. All measurements were carried out in duplicate. Analytical details regarding air sampling are provided in Table 2.

**Table 2:** Adsorbent media, analytical methods and sampling parameters

Substance group	Adsorber	Analysis	(normative) Reference	Air flow rate [mL min^−1^]	Sampling volume [L]
VVOC	Carbograph™ 5TD (20/40 mesh)	TD-GC-MS	Schieweck et al. [21]	125	4 (stage 1 + 2) 0.5 (stage 3)
VOC	Tenax® TA (60/80 mesh)	TD-GC-MS	ISO 16000–6 [22]	125	4 (stage 1 + 2) 0.5 (stage 3)
VOC	Tenax® TA (60/80 mesh)	GC-O	−	1000	8 (stage 1 + 2) 1 (stage 3)

#### Sampling very volatile organic compounds (VVOCs)

For trapping VVOCs single-bed stainless steel desorption tubes (Markes International Ltd., 89 mm length, 6.4 mm O.D.) were used which were filled with Carbograph™ 5TD (20/40 mesh, Markes International Ltd.), a graphitized carbon black (GCB). Body bag air was sampled actively by means of a pump with a flow rate of 125 mL min^−1^ and a total sampling volume of 0.5 L to 4 L in dependence of the decomposition stage. Corpses with advanced decomposition (stage 3) are expected to release much higher concentrations of gaseous contaminants, which might lead to a saturation of the solid sorbent. This would complicate both the identification and quantification of substances trapped on the sorbent. After sampling, the tubes were sealed with Swagelok brass end caps fitted with PTFE ferrules. For analysis, the tubes were thermally desorbed (300 °C, TD-100, Markes International Ltd.) into a coupled GC-MS system (Agilent 7890A/5975C). The conditions for thermal desorption were prepurge 3 min with a flow rate of 50 mL min^−1^, primary desorption at 300 °C for 6 min at a flow rate of 20 mL min^−1^, no inlet split, cold trap low at 25 °C, pretrap fire purge 3 min at 50 mL min^−1^, trap heating rate 40 °C s^−1^, cold trap high at 300 °C for 6 min, outlet split 10 mL min^−1^, and the flow path temperature at 200 °C. The cold trap contained quartz wool/Carbograph™ 1TD (40/60 mesh) and Carboxen® 1000 (80/100 mesh) with a ratio of 1:4.

The compounds were separated on a fused silica capillary column (6%/94% cyanopropylphenyl/dimethylpolysiloxane) of medium polarity (DB 624, 60 m, 0.32 mm, 1.8 µm, Agilent J&W). The initial column oven temperature was 30 °C (6 min), increased in a first step at 45 °C (1 °C min^−1^) and in a second step to 240 °C with an increasing rate of 40 °C min^−1^. The GC was operated in scan mode with a mass range of 20–450 amu, the MS source temperature was 230 °C, and the quadrupole temperature was 150 °C.

Data were processed using ChemStation® software mass spectral library. Qualifying was based on PBM library search. Mass spectra and retention data were compared with those of pure reference compounds. All identified substances were quantified using their own response factor. In dependence of the specific substance the limit of quantitation (LOQ) was ≤ 8 µg m^−3^. For analytical details and validation of the method it is referred to Schieweck et al. [21]. In the present study, VVOCs are defined as substances ≤ C_6_ which elute before n-hexane (C_6_) on a nonpolar GC column according to ISO 16000-6 [22] and which are not defined as VOCs according to EN 16516 [23], annex G, even though they are eluting before C_6_. A comprehensive discussion of different approaches for defining the term VVOCs has been published by Salthammer [24].

#### Sampling volatile organic compounds (VOCs)

VOCs were sampled on stainless steel desorption tubes (Markes International Ltd., 89 mm length, 6.4 mm O.D.) filled with the polymeric sorbent Tenax® TA (60/80 mesh, Chrompack). The air samples were drawn actively with a flow rate of 125 mL min^−1^. Due to the reasons outlined above (see Sect. "Sampling very volatile organic compounds (VVOCs)"), the total air volume differed between 0.5 L and 4 L. The tubes were subsequently analysed by GC-MS (Agilent 7890A/5975C) after thermal desorption (290 °C, 8 min; TD-100, Markes International Ltd.) in accordance with ISO 16000-6 [22]. The cold trap contained Carbograph™ 2/Carbograph™ 1 (Markes International Ltd.), cold trap low -25 °C. The transfer line temperature was 180 °C. Separation was performed on a nonpolar DB-5 MS column (5%/95% diphenyl/dimethylpolysiloxane, 60 m × 0.25 mm, 0.25 µm) using a starting temperature of 32 °C, followed by a 5 °C min^−1^ ramp to 150 °C, and a final 10 °C min^−1^ ramp to 300 °C. The MS was operated in scan mode with a mass range of 25–550 amu, MS source temperature of 250 °C, and quadrupole temperature of 150 °C.

Mass spectra and retention data were identified with those of pure reference compounds. For quantification, the own response factors were used. Limit of quantitation (LOQ) was ≤ 1 µg m^−3^. The TVOC value is defined as the value of total volatile organic compounds given as the total response of identified and unidentified substances which elute within the retention range between n-hexane (C_6_) and n-hexadecane (C_16_) [22].

Measurement results of acetic acid obtained by the use of Tenax® TA are included in this study, even though it is well-known that these are not reliable as the sampling volume in body bags was too small to allow a further, much more precise determination of C_1_-C_2_ carboxylic acids [21].

#### Odour analysis of air samples using GC-O

Odour analysis was performed using gas chromatography-olfactometry (GC-O) coupled with flame ionisation detection (FID). GC-O is an additional tool in order to identify odour-related substances, which cannot be detected by routine GC-MS analysis. After thermal desorption of Tenax® TA tubes (300 °C; Unity 2, Markes International Ltd.), substances were separated and analysed by GC-O/FID (Agilent 7890) using a nonpolar HP-5 MS column (5%/95% diphenyl/dimethylpolysiloxane, 30 m × 0.25 mm × 0.25 µm). The carrier gas (helium) of the TD-GC-O/FID system had a constant pressure of 1 bar resulting in a flow of about 1.38 mL min^−1^ at 35 °C (calculated). At the end of the GC column the gas flow was divided with a Y-splitter into two parts. One part flowed into the olfactory detection port (ODP 3, Gerstel), the other part into the FID (ratio 2:1).

The sniffing of the effluent was done by two well-trained and experienced evaluators. The run of the analysis was limited to about 30 min in order to avoid fatigue. The evaluators marked the odour active substances individually by use of a voice recognition software (Dragon NaturalSpeaking 10.0) and described the odour quality and intensity in single words, such as e.g. fruity, sweet, floral, pungent or sour. The identification of the resolved odourants based on the odour perception, in-house retention index library and prevalent in comparison to data obtained by GC-MS analysis. The retention indices (RI) were calculated with standardized parameters in accordance to the definition by van den Dool and Kratz [25]. The same was done to estimate the retention indices of the odourous substances found in the samples, using n-alkanes as external references. RI values were compared with RIs from compilations. If no reference standard was available, these compilations were used for identification.

The intensities were directly evaluated by means of a four-stage scale after plausibility check and averaging: (1) uncertain/very weak, (2) weak, (3) significant, (4) strong.

#### Emission testing of body bags

In order to separate emissions of the corpses from those released by the body bags themselves, single emission testing of the bag materials is needed as they might act as significant emission sources. Therefore, small pieces were cut off from the body bags which were subjected to emission testing in a 23 L emission test chamber that fulfils the specifications of ISO 16000-9 [26]. Testing conditions were 23 ± 1 °C temperature, 50 ± 5% relative humidity and 0.5 h^−1^ air exchange rate. Loading factor was approximately 0.7 m^2^ m^−3^. Active air sampling was performed on VVOCs and VOCs, 5 h and 24 h after starting chamber testing.

## Results and discussion

### Emissions from body bags (blank values)

**Fig. 1:**
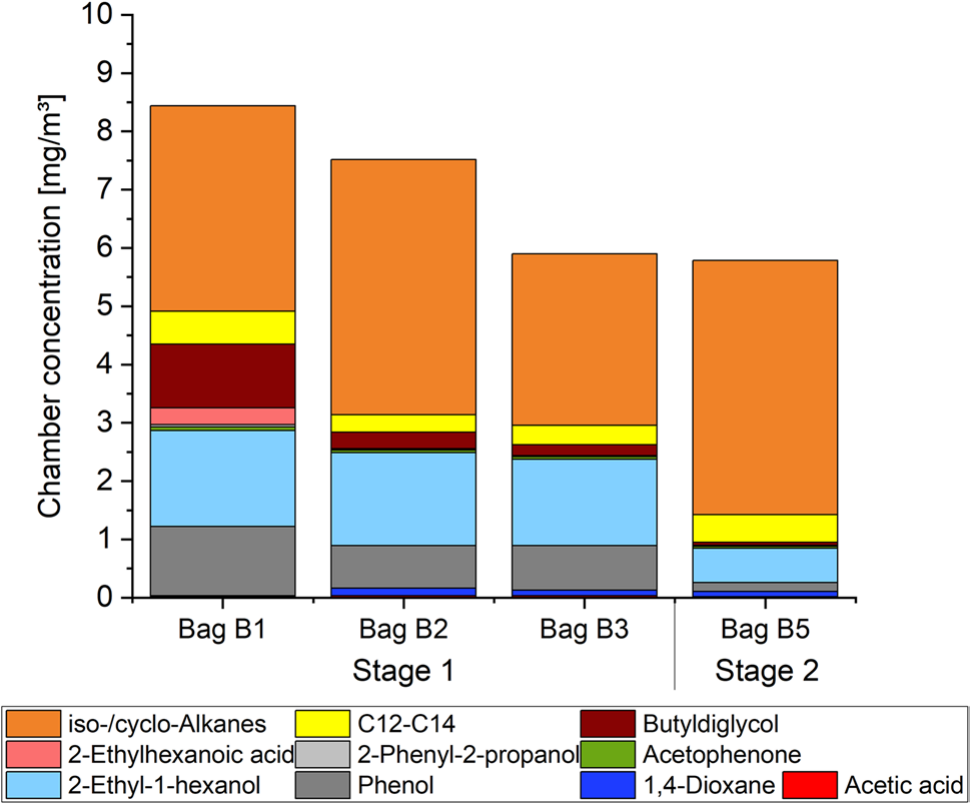
T(V)VOC-values (total (very) volatile organic compounds) obtained by sampling body bag air and room air. Bag Bx: Body bag of body number x

In total, emissions of four body bags were tested. Short term testing was sufficient in order to reveal information about the emission potential and released main compounds of the body bags, as illustrated in Fig. 1. Even though the bags differed in material and colour, the spectrum of released substances was nearly the same with just differing concentrations. A variety of iso-alkanes and cyclo-alkanes was emitted as main substance group with levels ranging between approximately 3 mg m^−3^ and 4.5 mg m^−3^ after 24 h testing time. In addition, 2-ethyl-1-hexanol and phenol were released in high concentrations. Also, 1,4-dioxane was identified in concentrations of up to 133 µg m^−3^ and might be used as solvent during the production of tissue cleaners, colorants and degreasing agents.

### Emissions from corpses

**Fig. 2:**
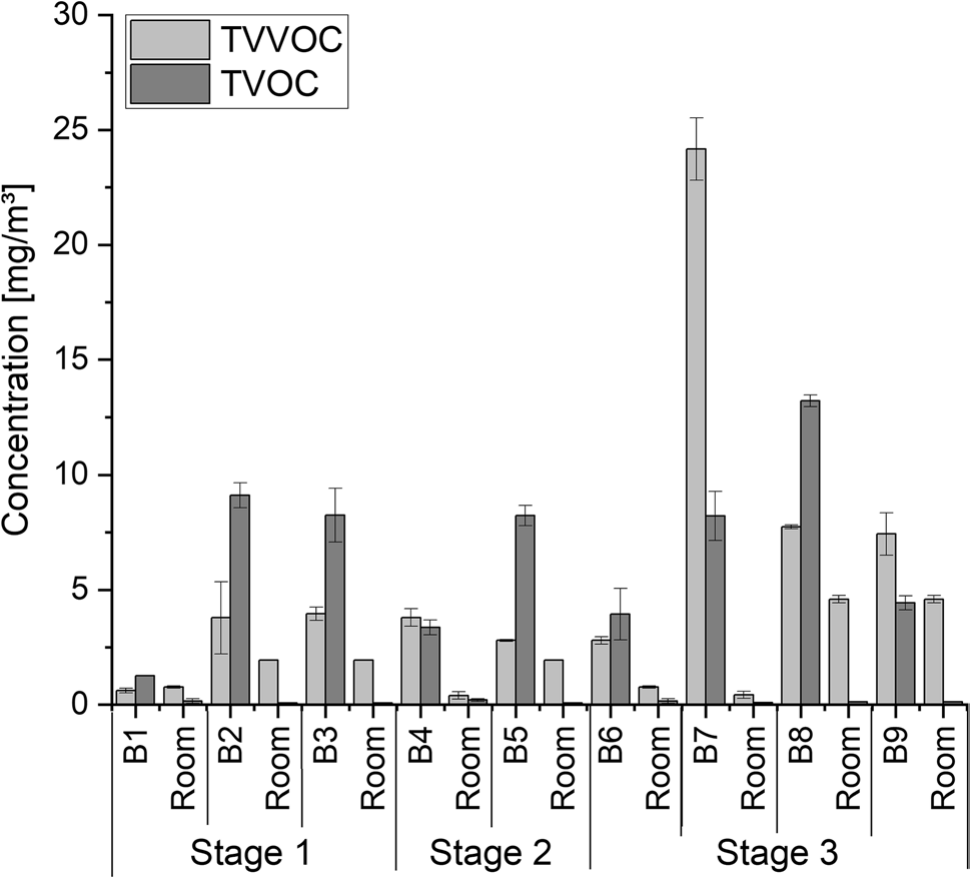
Most abundant substances and substance groups released by body bags. Bx: Body number x, Room: Autopsy room

Figure 2 provides an overview of the obtained sum concentrations of VVOCs and VOCs (T(V)VOC, total (very) volatile organic compounds). Detected concentration levels were highest in decomposition stage 3, but varied from body to body in all stages with no clear dependence on the decomposition stage. Concentrations of both VVOCs and VOCs were much lower in room air than in body bag air. Table 3 lists all identified substances emitted from deceased persons in different decomposition stages.

**Table 3:** Detected substances emitted from deceased persons in different decomposition stages given as arithmetic mean ± standard deviation

CAS No.	Substance name	Decomposition stage		
		1: Fresh (*N* = 3)	2: Initial (*N* = 2)	3: Advanced (*N* = 4)
		Concentration [µg m^−3^] (N)		
Urea derivatives				
*57-13-6*	*Urea*	n.d.	n.d.	14 ± 3 (1)
Alkanes				
*75-28-5*	*2-Methylpropane*	n.d.	2 ± 1 (1)	15 ± 11 (2)
*115-11-7*	*2-Methylpropene*	n.d.	n.d.	15 ± 4 (1)
*106-97-8*	*Butane*	n.d.	**4 ± 2 (2)**	34 ± 15 (1)
*78-78-4*	*2-Methylbutane*	5 ± 2 (1)	**4 ± 2 (2)**	20 ± 27 (2)
*109-66-0*	*n-Pentane*	5 ± 3 (1)	7 ± 1 (1)	47 ± 25 (1)
*107-83-5*	*2-Methylpentane*	5 ± 1 (1)	3 ± 1 (1)	n.d.
110-54-3	n-Hexane (C6)	n.d.	n.d.	16 ± 1 (1)
565-59-3	2,3-Dimethylpentane	n.d.	n.d.	4 ± 0 (1)
589-34-4	3-Methylhexane	4 ± 1 (2)	**2 ± 1 (2)**	11 ± 14 (2)
142-82-5	n-Heptane (C7)	**6 ± 2 (3)**	**5 ± 4 (2)**	40 ± 34 (3)
2613-61-8	2,4,6-Trimethylheptane	n.d.	n.d.	169 ± 175 (1)
592-13-2	2,5-Dimethylhexane	n.d.	n.d.	9 ± 2 (1)
589-43-5	2,4-Dimethylhexane	n.d.	n.d.	14 ± 4 (1)
584-94-1	2,3-Dimethylhexane	n.d.	n.d.	8 ± 1 (1)
1069-53-0	2,3,5-Trimethylhexane	n.d.	n.d.	3 ± 1 (1)
592-27-8	2-Methylheptane	n.d.	n.d.	40 ± 9 (1)
589-81-1	3-Methylheptane	4 ± 1 (2)	2 ± 0 (1)	35 ± 7 (1)
589-53-7	4-Methylheptane	n.d.	n.d.	15 ± 2 (1)
111-65-9	n-Octane (C8)	n.d.	n.d.	39 ± 40 (2)
1632-16-2	2-Ethyl-1-hexene	154 ± 1 (1)	149 ± 9 (1)	n.d.
2213-23-2	2,4-Dimethylheptane	16 ± 1 (1)	2 ± 1 (1)	**7 ± 3 (4)**
3074-75-7	4-Ethyl-2-methylhexane	n.d.	n.d.	3 ± 1 (1)
1072-05-5	2,6-Dimethyl-heptane	n.d.	n.d.	9 ± 1 (1)
16747-30-1	2,4,4-Trimethylhexane	n.d.	n.d.	4 ± 1 (1)
3074-71-3	2,3-Dimethylheptane	n.d.	1 ± 1 (1)	3 ± 1 (1)
3221-61-2	2-Methyloctane	n.d.	n.d.	23 ± 3 (1)
2216-33-3	3-Methyloctane	n.d.	n.d.	30 ± 4 (1)
2216-34-4	4-Methyloctane	10 ± 7 (2)	**3 ± 1 (2)**	11 ± 10 (3)
111-84-2	n-Nonane (C9)	4 ± 0 (1)	**11 ± 3 (2)**	**18 ± 24 (4)**
15869-89-3	2,5-Dimethyloctane	n.d.	n.d.	3 ± 0 (1)
13475-82-6	2,2,4,6,6-Pentamethylheptane	25 ± 19 (2)	**47 ± 39 (2)**	**35 ± 22 (4)**
124-18-5	n-Decane (C10)	**103 ± 88 (3)**	**39 ± 21 (2)**	**12 ± 6 (4)**
17302-37-3	2,2-Dimethyldecane	n.d.	29 ± 1 (1)	n.d.
2847-72-5	4-Methyldecane	11 ± 1 (1)	11 ± 0 (1)	n.d.
13151-35-4	5-Methyldecane	12 ± 2 (1)	13 ± 0 (1)	n.d.
17302-28-2	2,6-Dimethylnonane	n.d.	n.d.	13 ± 1 (2)
17302-32-2	3,7-Dimethylnonane	12 ± 2 (1)	15 ± 0 (1)	n.d.
7154-80-5	3,3,5-Trimethylheptane	n.d.	n.d.	25 ± 5 (1)
1120-21-4	n-Undecane (C11)	**96 ± 63 (3)**	**68 ± 61 (2)**	14 ± 7 (3)
112-40-3	n-Dodecane (C12)	**86 ± 47 (3)**	**99 ± 96 (2)**	**19 ± 10 (4)**
629-50-5	n-Tridecane (C13)	46 ± 31 (2)	222 ± 21 (1)	2 ± 0 (1)
629-59-4	n-Tetradecane (C14)	**16 ± 12 (3)**	**36 ± 40 (2)**	6 ± 1 (2)
629-62-9	n-Pentadecane (C15)	9 ± 6 (2)	17 ± 12 (2)	n.d.
544-76-3	n-Hexadecane (C16)	3 ± 3 (2)	8 ± 0 (1)	2 ± 0 (1)
Cycloalkanes				
110-82-7	Cyclohexane	n.d.	n.d.	2 ± 1 (2)
108-87-2	Methylcyclohexane	4 ± 1 (2)	3 ± 0 (1)	83 ± 18 (1)
2007-03-6	trans-1,3-Dimethylcyclohexane	6 ± 0 (1)	27 ± 11 (1)	36 ± 6 (1)
589-90-2	1,4-Dimethylcyclohexane	3 ± 0 (1)	n.d.	11 ± 1 (1)
2207-01-4	cis-1,2-Dimethylcyclohexane	n.d.	n.d.	11 ± 1 (1)
1795-27-3	cis-1,3,5-Trimethylcyclohexane	n.d.	n.d.	17 ± 3 (1)
1678-91-7	Ethylcyclohexane	4 ± 1 (2)	4 ± 0 (1)	17 ± 1 (1)
7667-60-9	1,2,4-Trimethylcyclohexane (1.alpha.,2.beta.,4.beta.)	n.d.	n.d.	21 ± 2 (1)
1839-63-0	1,3,5-Trimethylcyclohexane	n.d.	n.d.	10 ± 1 (1)
2234-75-5	1,2,4-Trimethylcyclohexane	n.d.	n.d.	42 ± 4 (1)
3728-55-0	1-Ethyl-3-methylcyclohexane	n.d.	n.d.	5 ± 1 (1)
1678-92-8	Propylcyclohexane	8 ± 4 (2)	6 ± 0 (1)	4 ± 0 (1)
1678-93-9	Butylcyclohexane	169 ± 180 (2)	8 ± 0 (1)	n.d.
493-02-7	trans-Decaline	19 ± 8 (2)	29 ± 1 (1)	n.d.
4390-04-9	2,2,4,4,6,8,8-Heptamethylnonane	13 ± 0 (1)	n.d.	n.d.
	Other cyclo-/iso-alkanes, sum	2467 ± 2009 (3)*	2460 ± 2201 (2)*	**559 ± 153 (4)**
Alkenes				
*78-79-5*	*Isoprene*	3 ± 0 (3)	8 ± 2 (2)	28 ± 18 (4)
19780-68-8	3-Ethyl-4-methyl-2-pentene	53 ± 6 (1)	48 ± 1 (1)	n.d.
7145-23-5	2,3-Dimethyl-3-hexene	140 ± 6 (1)	137 ± 10 (1)	n.d.
592-76-7	1-Heptene	n.d.	n.d.	10 ± 3 (1)
111-66-0	1-Octene	n.d.	3 ± 0 (1)	7 ± 1 (1)
3404-75-9	3-Methyl-2-heptene	187 ± 11 (1)	192 ± 9 (1)	n.d.
110-93-0	6-Methyl-5-hepten-2-one	n.d.	20 ± 1 (1)	n.d.
922-63-4	2-Ethylacrolein	2 ± 0 (1)	2 ± 0 (1)	19 ± 4 (1)
149196-01-8	trans-3-Octene	29 ± 1 (1)	28 ± 1 (1)	n.d.
2198-23-4	4-Nonene	13 ± 0 (1)	n.d.	n.d.
112-41-4	1-Dodecene	5 ± 0 (1)	n.d.	7 ± 4 (3)
Aldehydes				
*107-02-8*	*2-Propenal (Acrolein)*	5 ± 3 (1)	2 ± 0 (1)	n.d.
*107-22-2*	*Oxaldehyde (Glyoxal)*	n.d.	n.d.	20,222 ± 2142 (1)*
*123-38-6*	*Propanal*	13 ± 6 (1)	**10 ± 4 (2)**	n.d.
*78-85-3*	*2-Methylprop-2-enal (Methacrolein)*	n.d.	3 ± 1 (1)	53 ± 13 (1)
*123-72-8*	*Butanal*	**8 ± 5 (3)**	7 ± 0 (1)	n.d.
*78-84-2*	*2-Methylpropanal*	n.d.	n.d.	150 ± 37 (1)
*590-86-3*	*3-Methylbutanal*	6 ± 1 (2)	**7 ± 4 (2)**	**87 ± 44 (4)**
*4170-30-3*	*Crotonaldehyde*	n.d.	**7 ± 3 (2)**	38 ± 8 (1)
*123-73-9*	*trans-Crotonaldehyde (trans-2-Butenal)*	n.d.	n.d.	2 ± 1 (1)
*107-86-8*	*3-Methyl-2-butenal*	n.d.	3 ± 0 (1)	n.d.
*110-62-3*	*Pentanal*	n.d.	11 ± 0 (1)	n.d.
66-25-1	Hexanal	**36 ± 15 (3)**	**45 ± 12 (2)**	n.d.
123-05-7	2-Ethylhexanal	30 ± 10 (2)	**21 ± 5 (2)**	n.d.
111-71-7	Heptanal	**6 ± 1 (3)**	8 ± 0 (1)	4 ± 1 (1)
100-52-7	Benzaldehyde	3 ± 1 (1)	3 ± 0 (1)	**11 ± 7 (4)**
124-13-0	Octanal	6 ± 1 (1)	10 ± 0 (1)	9 ± 3 (2)
124-19-6	Nonanal	31 ± 2 (1)	33 ± 1 (1)	n.d.
112-31-2	Decanal	8 ± 0 (1)	6 ± 1 (1)	n.d.
112-54-9	Dodecanal	2 ± 0 (1)	n.d.	3 ± 1 (1)
Ketones				
*67-64-1*	*2-Propanone (Acetone)*	**171 ± 40 (3)**	**358 ± 58 (2)**	1187 ± 632 (3)
*431-03-8*	*2,3-Butanedione*	6 ± 3 (2)	**18 ± 19 (2)**	28 ± 23 (2)
*78-93-3*	*2-Butanone (MEK; Methyl ethyl ketone)*	**31 ± 3 (3)**	**29 ± 8 (2)**	**242 ± 246 (4)**
*563-80-4*	*3-Methyl-2-butanone (MIPK; Methyl isopropyl ketone)*	n.d.	8 ± 1 (1)	19 ± 0 (1)
108-10-1	4-Methylpentan-2-one (MIBK; Methyl isobutyl ketone)	15 ± 9 (2)	4 ± 0 (1)	n.d.
*107-87-9*	*2-Pentanone*	**8 ± 1 (3)**	**8 ± 1 (2)**	**118 ± 92 (4)**
*96-22-0*	*3-Pentanone*	n.d.	n.d.	4 ± 0 (1)
*116-09-6*	*1-Hydroxy-2-propanone*	n.d.	n.d.	3 ± 3 (1)
*513-86-0*	*3-Hydroxy-2-butanone (Acetoin)*	27 ± 1 (1)	31 ± 4 (1)	71 ± 46 (2)
*78-94-4*	*Methyl vinyl ketone*	n.d.	n.d.	28 ± 3 (1)
565-61-7	3-Methyl-2-pentanone	n.d.	n.d.	4 ± 1 (1)
1120-72-5	2-Methylcyclopentanone	n.d.	n.d.	285 ± 14 (1)
591-78-6	2-Hexanone	n.d.	1 ± 0 (1)	5 ± 3 (2)
589-38-8	3-Hexanone	n.d.	n.d.	2 ± 0 (1)
110-43-0	2-Heptanone	n.d.	4 ± 0 (1)	15 ± 18 (3)
106-35-4	3-Heptanone	8 ± 0 (1)	1 ± 0 (1)	n.d.
108-94-1	Cyclohexanone	8 ± 4 (2)	n.d.	n.d.
110-93-0	6-Methyl-5-hepten-2-one	6 ± 0 (1)	n.d.	12 ± 17 (2)
106-68-3	3-Octanone	n.d.	n.d.	4 ± 0 (1)
98-86-2	Acetophenone	42 ± 2 (2)	**14 ± 15 (2)**	**5 ± 3 (4)**
821-55-6	2-Nonanone	n.d.	n.d.	4 ± 1 (1)
719-22-2	2,6-Di-tert-butyl-1,4-benzoquinone	2 ± 0 (1)	n.d.	n.d.
Alcohols				
*64-17-5*	*Ethanol*	**886 ± 1126 (3)**	2173 ± 811 (2)*	**281 ± 88 (4)**
*71-23-8*	*1-Propanol (n-Propanol)*	**138 ± 85 (3)**	222 ± 1 (1)	241 ± 144 (2)
*67-63-0*	*2-Propanol*	200 ± 14 (2)	481 ± 4 (1)	**220 ± 316 (4)**
*78-83-1*	*Isobutanol*	**15 ± 14 (3)**	**9 ± 1 (2)**	**475 ± 688 (4)**
*71-36-3*	*1-Butanol*	**26 ± 13 (3)**	**211 ± 218 (2)**	263 ± 315 (3)
*78-92-2*	*2-Butanol*	2 ± 0 (1)	4 ± 0 (1)	**19 ± 15 (4)**
*123-51-3*	*3-Methyl-1-butanol*	**8 ± 3 (3)**	**14 ± 4 (3)**	1279 ± 2113 (4)*
*598-75-4*	*3-Methyl-2-butanol*	n.d.	n.d.	17 ± 1 (1)
*616-25-1*	*1-Penten-3-ol*	2 ± 0 (1)	**2 ± 0 (2)**	n.d.
*57-55-6*	*1,2-Propanediol*	99 ± 100 (2)	6 ± 2 (1)	n.d.
*71-41-0*	*1-Pentanol*	n.d.	10 ± 1 (1)	**14 ± 12 (4)**
*6032-29-7*	*2-Pentanol*	n.d.	n.d.	9 ± 2 (2)
626-89-1	4-Methyl-1-pentanol	n.d.	n.d.	12 ± 1 (1)
123-44-4	2,2,4-Trimethyl-1-pentanol	79 ± 6 (1)	146 ± 4 (1)	n.d.
104-76-7	2-Ethyl-1-hexanol	**1048 ± 766 (3)**	**301 ± 341 (2)**	26 ± 13 (2)
617-94-7	2-Phenyl-2-propanol	21 ± 2 (2)	16 ± 6 (1)	n.d.
60-12-8	2-Phenylethanol	n.d.	n.d.	2 ± 1 (3)
112-42-5	Undecanol	n.d.	n.d.	78 ± 15 (1)
Aromatic hydrocarbons				
71-43-2	Benzene	4 ± 1 (2)	**3 ± 1 (2)**	34 ± 23 (2)
108-88-3	Toluene	**33 ± 22 (3)**	**10 ± 5 (2)**	**37 ± 9 (4)**
100-41-4	Ethylbenzene	7 ± 1 (2)	5 ± 0 (1)	4 ± 0 (1)
1330-20-7	m, p-Xylene	**28 ± 5 (3)**	**14 ± 5 (2)**	**16 ± 4 (4)**
100-42-5	Styrene	1 ± 1 (3)	4 ± 0 (1)	1 ± 1 (2)
95-47-6	o-Xylene	**10 ± 0 (3)**	6 ± 1 (1)	3 ± 1 (3)
98-82-8	Isopropylbenzene (Cumene)	21 ± 2 (2)	33 ± 1 (1)	n.d.
576-26-1	2,6-Dimethylphenol	n.d.	2 ± 0 (1)	n.d.
108-95-2	Phenol	**481 ± 376 (4)**	**154 ± 38 (3)**	**239 ± 417 (4)**
98-83-9	α-Methylstyrene	10 ± 2 (2)	n.d.	n.d.
95-63-6	1,2,4-Trimethylbenzene	2 ± 0 (1)	1 ± 0 (1)	1 ± 0 (3)
106-46-7	1,4-Dichlorobenzene	n.d.	3 ± 0 (1)	n.d.
535-77-3	m-Cymene	n.d.	n.d.	13 ± 9 (2)
496-11-7	Indan	n.d.	n.d.	2 ± 0 (1)
106-44-5	p-Cresol	n.d.	n.d.	12 ± 1 (1)
	Naphthalene derivatives, sum	**43 ± 31 (3)**	98 ± 53 (1)	2 ± 0 (1)
65-85-0	Benzoic acid	n.d.	n.d.	24 ± 17 (3)
96-76-4	2,4-Di-tert-butylphenol	1 ± 0 (2)	n.d.	2 ± 1 (2)
128-37-0	Dibutylhydroxytoluene (BHT)	64 ± 35 (1)	n.d.	n.d.
	Other C3-benzenes, sum	11 ± 0 (1)	n.d.	n.d.
	Other C4-benzenes, sum	n.d.	n.d.	14 ± 1 (1)
Carboxylic acids				
*64-19-7*	*Acetic acid*	**77 ± 73 (3)**	**44 ± 32 (2)**	39 ± 29 (2)
*79-09-4*	*Propionic acid*	n.d.	n.d.	188 ± 37 (1)
*107-92-5*	*Butanoic acid*	n.d.	37 ± 8 (1)	n.d.
*109-52-4*	*Valeric acid*	n.d.	n.d.	3 ± 3 (1)
*503-74-2*	*Isovaleric acid*	n.d.	n.d.	14 ± 3 (1)
124-07-2	Octanoic acid	2 ± 0 (1)	n.d.	n.d.
57-10-3	Palmitic acid	n.d.	n.d.	157 ± 30 (1)
112-80-1	Oleic acid	n.d.	n.d.	19 ± 1 (1)
57-11-4	Stearic acid	n.d.	n.d.	15 ± 4 (1)
Carboxylic acid esters				
*109-94-4*	*Ethyl formate*	n.d.	n.d.	68 ± 12 (1)
*592-84-7*	*Butyl formate*	6 ± 0 (1)	n.d.	n.d.
*79-20-9*	*Methyl acetate*	2 ± 0 (1)	5 ± 1 (1)	n.d.
*141-78-6*	*Ethyl acetate*	11 ± 5 (2)	6 ± 0 (1)	n.d.
*109-60-4*	*Propyl acetate*	n.d.	12 ± 1 (1)	n.d.
97-62-1	Ethyl isobutyrate	4 ± 0 (1)	n.d.	n.d.
105-54-4	Ethyl butyrate	39 ± 1 (1)	n.d.	17 ± 2 (1)
123-86-4	n-Butyl acetate	4 ± 2 (2)	**3 ± 1 (2)**	4 ± 0 (1)
105-46-4	sec-Butyl acetate	4 ± 0 (1)	2 ± 1 (1)	n.d.
110-19-0	Isobutyl acetate	n.d.	n.d.	2 ± 0 (1)
7452-79-1	Ethyl 2-methylbutyrate	4 ± 1 (1)	n.d.	n.d.
108-64-5	Ethyl isovalerate	3 ± 0 (1)	n.d.	n.d.
123-92-2	Isopentyl acetate	n.d.	3 ± 1 (1)	11 ± 9 (2)
624-41-9	2-Methylbutyl acetate	n.d.	n.d.	3 ± 1 (1)
123-66-0	Ethyl hexanoate	n.d.	n.d.	10 ± 0 (1)
109-21-7	Butyl butyrate	3 ± 0 (1)	n.d.	12 ± 1 (1)
103-09-3	2-Ethylhexyl acetate	n.d.	2 ± 0 (1)	n.d.
Ethers				
107-98-2	Propylene glycol methyl ether (PGME, 1-methoxy-2-propanol)	6 ± 0 (1)	4 ± 0 (1)	25 ± 2 (1)
105-57-7	1,1-Diethoxyethane	n.d.	n.d.	3 ± 0 (1)
111-76-2	2-Butoxyethanol	**7 ± 4 (3)**	**3 ± 2 (2)**	3 ± 1 (3)
112-34-5	Diethylene glycol butyl ether (butyldiglycol)	**157 ± 131 (3)**	50 ± 22 (1)	7 ± 3 (2)
122-99-6	2-Phenoxyethanol	6 ± 0 (1)	n.d.	**7 ± 6 (4)**
Halogenated ethers				
*28523-86-6*	*Sevoflurane*	206 ± 89 (1)	n.d.	n.d.
Chlorinated hydrocarbons				
*75-71-8*	*Dichlorodifluoromethane*	n.d.	8 ± 0 (1)	n.d.
*594-37-6*	*1,2-Dichloro-2-methylpropane*	n.d.	n.d.	16 ± 11 (1)
*75-09-2*	*Dichloromethane*	1 ± 0 (2)	n.d.	n.d.
*109-69-3*	*1-Chlorobutane*	31 ± 3 (2)	21 ± 1 (1)	n.d.
*67-66-3*	*Trichloromethane (Chloroform)*	1 ± 0 (1)	n.d.	3 ± 1 (2)
*78-87-5*	*1,2-Dichloropropane*	48 ± 28 (2)	14 ± 1 (1)	n.d.
Sulfurous compounds				
*74-93-1*	*Methanethiol*	n.d.	n.d.	4 ± 3 (1)
*75-15-0*	*Carbon disulfide*	n.d.	4 ± 0 (1)	41 ± 28 (2)
*75-18-3*	*Dimethyl sulfide (DMS)*	11 ± 8 (2)	14 ± 1 (1)	27 ± 6 (1)
*624-92-0*	*Dimethyl disulfide (DMDS)*	**317 ± 48 (3)**	**863 ± 489 (2)**	1621 ± 697 (4)*
*20333-39-5*	*Methyl ethyl disulfide*	n.d.	n.d.	2 ± 1 (1)
*40136-65-0*	*Methyl isopropyl disulfide*	n.d.	n.d.	12 ± 1 (1)
*67-71-0*	*Dimethyl sulfone*	n.d.	n.d.	2 ± 1 (1)
*556-64-9*	*Methyl thiocyanate*	n.d.	n.d.	139 ± 69 (1)
*556-61-6*	*Methyl isothiocyanate*	n.d.	n.d.	210 ± 170 (1)
*3658-80-8*	*Dimethyl trisulfide (DMTS)*	23 ± 13 (2)	**124 ± 120 (2)**	**720 ± 702 (4)**
*5756-24-1*	*Dimethyl tetrasulfide*	2 ± 0 (1)	5 ± 1 (1)	132 ± 103 (2)
42474-44-2	2,3,4-Trithiahexane (Methyl (methylthio) methyl disulfide)	n.d.	n.d.	4 ± 0 (1)
Nitrogenous compounds				
*78-81-9*	*Isobutylamine*	n.d.	n.d.	53 ± 33 (1)
*75-50-3*	*Trimethylamine (TMA)*	57 ± 15 (2)	75 ± 6 (1)	1476 ± 1225 (4)*
*107-85-7*	*3-Methyl-1-butylamine*	n.d.	n.d.	284 ± 82 (1)
*107-13-1*	*Acrylonitrile*	n.d.	n.d.	6 ± 1 (1)
*75-05-8*	*Acetonitrile*	n.d.	8 ± 0 (1)	45 ± 9 (2)
100-47-0	Benzonitrile	n.d.	n.d.	4 ± 1 (1)
123-32-0	2,5-Dimethylpyrazine	n.d.	n.d.	8 ± 1 (1)
110-86-1	Pyridine	3 ± 2 (2)	3 ± 0 (1)	76 ± 77 (2)
*123-75-1*	*Pyrrolidine*	n.d.	n.d.	11 ± 1 (1)
*68-12-2*	*N,N-Dimethylformamide*	n.d.	n.d.	13 ± 2 (1)
926-64-7	(Dimethylamino) acetonitrile	n.d.	n.d.	254 ± 255 (2)
291-22-5	Quinoline	n.d.	n.d.	3 ± 0 (1)
120-72-9	Indole	n.d.	n.d.	17 ± 16 (3)
Terpenes				
80-56-8	alpha-Pinene	**38 ± 19 (3)**	**60 ± 33 (2)**	**386 ± 606 (4)**
79-92-5	Camphene	n.d.	1 ± 0 (1)	5 ± 6 (3)
18172-67-3	beta-Pinene	9 ± 6 (2)	**6 ± 4 (2)**	14 ± 13 (3)
123-35-3	beta-Myrcene	3 ± 1 (1)	n.d.	24 ± 23 (2)
498-15-7	3-Carene	**48 ± 27 (3)**	**58 ± 29 (2)**	348 ± 441 (3)
5989-27-5	Limonene	18 ± 8 (2)	**16 ± 13 (2)**	**31 ± 20 (4)**
1195-31-9	( +)-p-Menth-1-en	9 ± 1 (1)	12 ± 0 (1)	n.d.
3387-41-5	Sabinene	2 ± 1 (1)	n.d.	n.d.
470-82-6	Eucalyptol	8 ± 0 (1)	n.d.	6 ± 1 (1)
586-62-9	Terpinolene	4 ± 1 (1)	4 ± 0 (1)	12 ± 15 (3)
76-22-2	Camphor	7 ± 0 (1)	n.d.	2 ± 1 (2)
14073-97-3	L-menthan-3-one	n.d.	n.d.	2 ± 1 (1)
3623-51-6	( ±)-Neomenthol	n.d..	n.d.	2 ± 0 (1)
89-78-1	Menthol	n.d.	n.d.	28 ± 5 (1)
7785-53-7	alpha-Terpineol	n.d.	n.d.	4 ± 1 (1)
80-57-9	Verbenone	n.d.	n.d.	2 ± 0 (1)
	Other terpenes, unidentified, sum	n.d.	2 ± 0 (1)	13 ± 10 (3)
Phthalates				
131-11-3	Dimethyl phthalate	2 ± 0 (1)	n.d.	n.d.
Oxygen heterocyclics				
123-91-1	1,4-Dioxane	114±19 (2)	86±7 (1)	n.d.
Silanes/Silanols				
*1066-40-6*	*Trimethylsilanol*	3 ± 2 (2)	n.d.	n.d.
*353-66-2*	*Dimethyldifluorosilane*	n.d.	n.d.	5 ± 1 (1)
Siloxanes				
541-05-9	Hexamethylcyclotrisiloxane (D3)	**3 ± 1 (3)**	**11 ± 10 (2)**	**41 ± 13 (4)**
556-67-2	Octamethylcyclotetrasiloxane (D4)	3 ± 2 (2)	**19 ± 18 (2)**	43 ± 28 (3)
541-02-6	Decamethylcyclopentasiloxane (D5)	**4 ± 2 (3)**	**6 ± 3 (2)**	**11 ± 4 (4)**
540-97-6	Dodecamethylcyclohexasiloxane (D6)	n.d.	2 ± 1 (1)	2 ± 1 (2)
	Other siloxanes, unidentified, sum	9 ± 1 (1)	n.d.	**6 ± 7 (4)**

N = number of measurements (body bags), in which the substance was identified. VVOC-substances given in *italics*

^*^ An overload of the sampling tube can be assumed; therefore, an underestimation of the quantification results might be possible

**Fig. 3:**
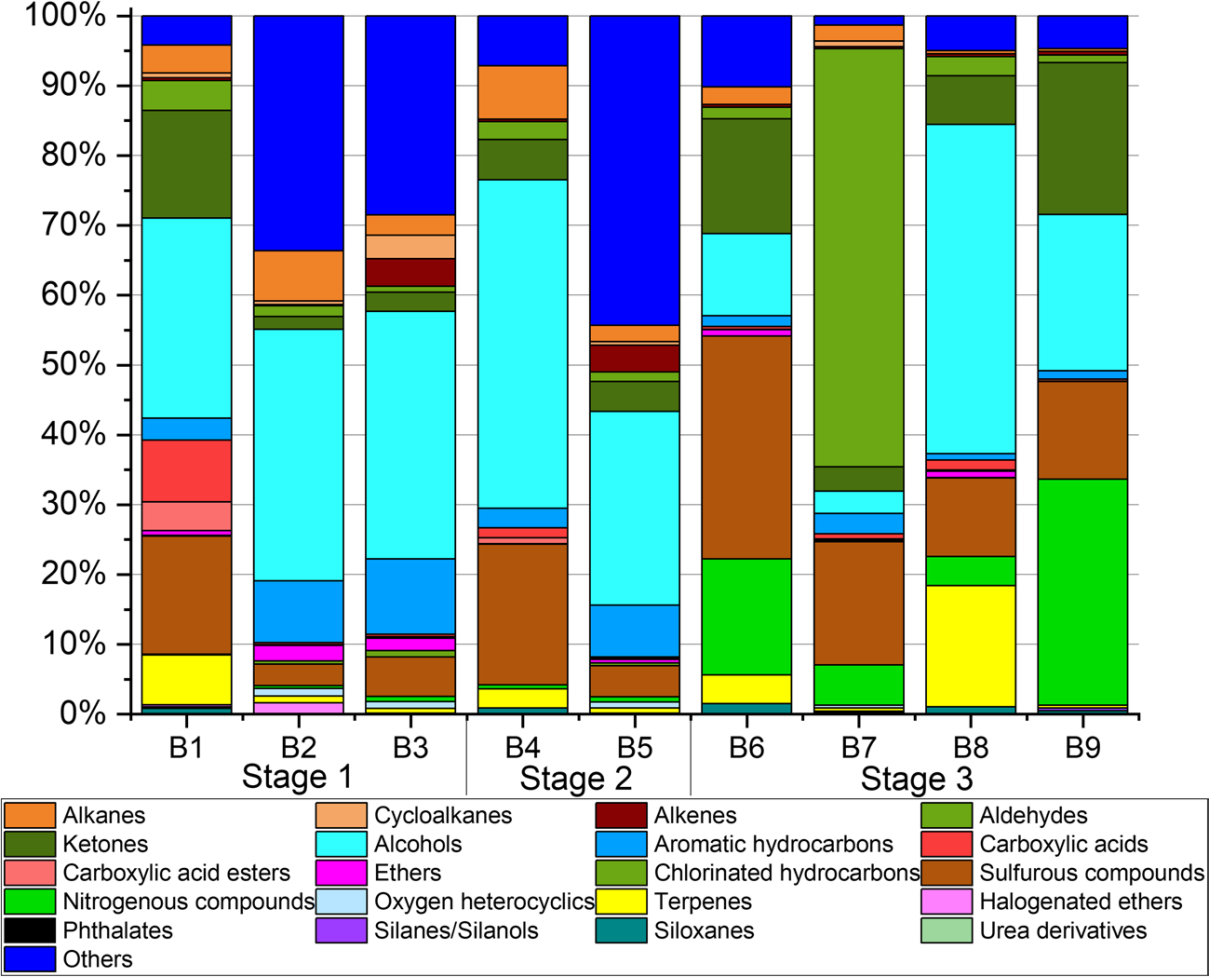
Most abundant substances and substance groups released by human bodies in dependence of the decomposition stage. Bx: Body number x

Most abundant substances identified in the environmental room air were alcohols (ethanol, 2-propanol) and glycol ethers (2-phenoxy ethanol, butyl glycol, butyldiglycol) which can be traced back to cleaning agents and disinfectants. In body bag air, around 350 individual organic substances were detected in total. Table 3 lists all substances which have been identified during this study by giving CAS-no, substance names, categorization as VVOC or VOC and the detected concentration range (arithmetic mean values ± standard deviation) within each decomposition stage as concentration in the body bags. If a clear identification was not possible, these compounds were summarized as a sum parameter of unidentified substances in the specific chemical group. Figure 3 illustrates the identified substance groups in each decomposition stage.

#### Fresh bodies

Three bodies with no visible signs of decomposition were selected for stage 1 (fresh bodies). In comparison, body no. 1 showed the lowest emission potential, which can be explained by the fact that the person was only recently passed away (1 d). TVVOC and TVOC-values were much higher regarding body no. 2 and no. 3 with 4 and 5 days since death. Both a broader range of identified substances and higher concentrations might be therefore be attributed to a longer PMI. Most abundant substance groups were alcohols (ethanol, 1-propanol, 2-propanol, 1,2-propanediol, 2-ethyl-1-hexanol) and n-alkanes. Some of these are attributed to the breakdown of sugars during early decomposition [1, 27]. However, pyruvic acid, which is described as an important intermediate product in the breakdown of carbohydrates and precursor substance for several alcohols, such as e.g. ethanol, butanol and 1,3-propanediol [1], was not found. Just 1,2-propanediol was emitted by bodies no. 2 and 3 (161 ± 39 µg m^−3^ and 13 ± 2 µg m^−3^) [1, 28]. Also, a variety of iso-/cyclic alkanes was measured, but could not be further identified. Very similar emission patterns and concentrations were detected for bodies no. 2 and 3 regarding the aldehydes n-pentanal, n-hexanal, 2-ethylhexanal and n-heptanal with concentrations between 6 µg m^−3^ and 48 µg m^−3^. Acetaldehyde was just detected for body no. 2 with the highest concentration within the group of aldehydes (62 ± 26 µg m^−3^). Already after a PMI of just 1 d (body no. 1), the alcohols ethanol and 2-propanol (254 ± 47 µg m^−3^ and 190 ± 1 µg m^−3^) and the ketone acetone (209 ± 4 µg m^−3^) were already detected as most abundant substances. Acetone is formed by microbial activity from carbohydrates under anaerobic conditions and is most frequently detected in exhaled air [29–31]. Similar acetone concentrations were determined for bodies no. 2 and 3. 3-Hydroxy-2-butanone (acetoin) was just identified as emission from body no. 1 (27 ± 1 µg m^−3^). It is released during the anaerobic glucose metabolism and as metabolic product of e.g. enterobacteria or lactic acid bacteria [32].

**Fig. 4:**
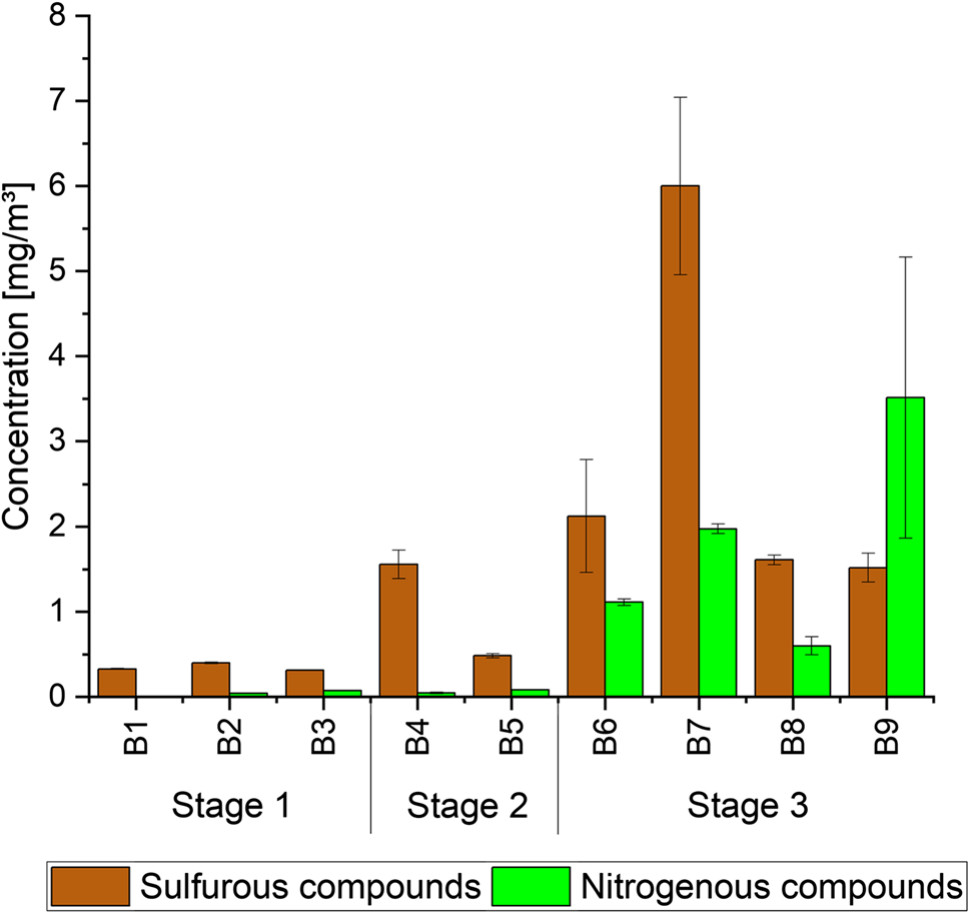
Concentrations of sulfurous and nitrogenous compounds released by deceased bodies. Bx: Body number x

Levels of ethanol and 2-propanol were in a similar range than acetone concentrations, whereas body no. 2 emitted ethanol in elevated concentrations of 2228 ± 967 µg m^−3^. Also, 2-ethyl-1-hexanol was released in high concentrations by bodies no. 2 and 3 (1596 ± 95 µg m^−3^ and 1484 ± 62 µg m^−3^). Moreover, both bodies emitted a broad range of aromatic hydrocarbons, namely benzene, toluene, ethylbenzene, xylene isomers, styrene, isopropylbenzene as well as methylstyrene derivatives and naphthalene derivatives, which could not be further identified. Highest levels were measured for phenol with 727 ± 141 µg m^−3^ (body no. 2) and 766 ± 20 µg m^−3^ (body no. 3). However, this can mainly be traced back to emissions of the body bags themselves. This also applied for 1,4-dioxane, which was just detected as emissions from body bags no. 2 and 3 (133 ± 11 µg m^−3^ and 96 ± 3 µg m^−3^). The occurrence of aromatic hydrocarbons is due to the breakdown of aromatic amino acids [15, 28]. Elevated concentrations of butylcyclohexane were analysed for bodies no. 2 and 3. In comparison, body no. 1 released a broad range of terpenes as listed in Table 3. Sabinene, myrcene, (alpha-) terpinolene, beta-pinene, eucalyptol, camphene and limonene were detected in just minor concentration (2 ± 1 µg m^−3^ to 11 ± 1 µg m^−3^). In contrast, alpha-pinene and 3-carene were identified in elevated concentrations (38 ± 1 µg m^−3^ and 60 ± 1 µg m^−3^, respectively). It is interesting to note that these terpene levels were detected for body no. 1, even though this person passed away just one day before the measurements. Alpha-pinene and limonene were also released in higher concentrations from body no. 2 (60 ± 1 µg m^−3^ and 70 ± 3 µg m^−3^, respectively). In addition, also acetic acid was released in significant concentrations (170 ± 23 µg m^−3^) from body no. 1, whereas the detected amounts were much lower in body bags of bodies no. 2 and 3 (32 ± 4 µg m^−3^ and 30 ± 11 µg m^−3^). Sulfurous and nitrogenous compounds are mainly associated with putrefaction [14]. Measured data are illustrated in Fig. 4. Even though the selected bodies showed a PMI of 1–5 d, dimethyl disulfide (DMDS) was detected in significant concentrations in all investigated body bags (289 ± 6 µg m^−3^ to 378 ± 14 µg m^−3^). DMDS is caused by oxidative reactions of methanethiol and hydrogen sulfide [1]. While methanethiol will not be trapped by Tenax® TA due to its low boiling point (5.9 °C), it can be sampled by Carbograph™ 5TD. Dimethyl trisulfide (DMTS) and dimethyl sulfide (DMS) as further oxidation products were just identified in levels in the range of the LOQ. No nitrogenous substances were analysed one day after death (body no. 1). Few days later, the odourous compound trimethylamine (TMA) was already detected in significant concentrations (bodies no. 2 and 3: 44 ± 1 µg m^−3^ and 71 ± 3 µg m^−3^, respectively). TMA is formed by dicarboxylic oxidation of proteins and can occur paired with dimethylamine (DMA) which was not determined [33]. As conspicuous finding, bodies no. 1 and 2 released sevoflurane, a narcotic agent. This was surprising since person no. 1 died a natural death. The person was neither reanimated nor intubated and was not under the influence of medication. Whereas detected concentrations were very low (7 ± 1 µg m^−3^), concentrations released by body no. 2 were significantly higher (247 ± 33 µg m^−3^). This person was both reanimated and intubated and died due to suicide (mixed intoxication). However, the use of sevoflurane for intubation is quite uncommon. Instead, lidocaine, an amino amide, is used.

### Initial decomposition

Even though the time since dead was similar to those corpses selected for stage 1, greenish discolouration in the lumbar region was already visible. As can be seen in Fig. 3, the most abundant substance groups were very similar to those identified for stage 1, namely n-alkanes, iso-/cyclo-alkanes, aromatic hydrocarbons, ketones, aldehydes and sulfurous substances. Thus, there are no clear differences between the emission characteristics of the corpses investigated in stages 1 and 2. In contrast to body no. 5, body no. 4 emitted carboxylic acids in moderate concentrations of 108 ± 27 µg m^−3^. Terpenes were released to a greater extent. Again, alpha-pinene and 3-carene were identified as most abundant substances, which is identical to the findings obtained for stage 1. It is interesting that a range of different naphthalene derivatives was released in elevated concentrations from body no. 5 (22-171 µg m^−3^) as a similar emission pattern was detected for bodies no. 2 and 3 (stage 1), but with lower concentration levels. However, those substances could not be identified to be released from body no. 4 even though it showed the same signs of decomposition than body no. 5. Among aromatic hydrocarbons, phenol was released as most abundant substance, but in significantly lower concentrations (157 ± 24 µg m^−3^ and 152 ± 62 µg m^−3^, respectively) than detected for the fresh bodies (stage 1). The same applied for 2-ethyl-1-hexanol, whose concentrations were lower by a factor of 2 regarding body no. 5, but could just be identified in trace concentrations in body bag air of body no. 4 (11 ± 1 µg m^−3^). Concentrations of ethanol were in the same range as those released by fresh bodies (stage 1). Regarding body no. 4, again acetoin was detected in low concentrations (31 ± 4 µg m^−3^).

However, due to initial signs of decomposition, one could have assumed that sulfurous and nitrogenous compounds will be emitted by the corpses in higher concentrations than by those with no visible decay (stage 1). However, this was just confirmed for body no. 4, which released dimethyl disulfide (DMDS) as main substance in high concentrations (1280 ± 155 µg m^−3^). Levels measured for body no. 5 were several factors lower with 447 ± 23 µg m^−3^. Dimethyl sulfide (DMS) and dimethyl trisulfide (DMTS) were identified as further sulfurous substances. Comparable to the findings obtained in stage 1, nitrogenous compounds were not released in significant concentrations. Again, trimethylamine (TMA) was identified but just in moderate concentrations (body no. 4: 42 ± 8 µg m^−3^ and body no. 5: 75 ± 6 µg m^−3^). Pyridine which is described in the literature to be one of the key substances to be emitted during the decomposition of humans and pigs [13, 16, 34] was detected in trace concentrations for body no. 4 (3 µg m^−3^), but could not be identified as emission from body no. 5.

### Advanced decomposition

A total of four persons with significant signs of advanced decay was emission tested. Body no. 7 was a very strong emission source. Aldehydes were identified as most abundant substance group in the body bag air due to glyoxal which was released in highly increased concentrations (20,222 ± 2141 µg m^−3^). Glyoxal is the smallest dialdehyde and can be used as a bio-marker to detect the development or progression of degenerative diseases, such as e.g. Alzheimer’s disease, chronic kidney disease and diabetes [35]. The occurrence in such high levels like those for body no. 7 cannot easily be explained. It can be assumed to be a degradation product of hydrocarbons or a combustion product of biomass. It also might be released by textiles where it is used to tear the strength of fibrous materials due to its ability to react with hydroxy and amino groups of proteins and cellulose [36]. Glyoxal was not detected in the other body bags of stage 3. Even though acetone was already identified as main substance in stages 1 and 2, it was released in concentrations by a factor of 10 higher from the advanced decomposed bodies no. 7 and 9 (3098 ± 10 µg m^−3^ and 1903 ± 557 µg m^−3^). The same applied for phenol (body no. 7: 906 ± 176 µg m^−3^) which, however, might be evaluated as background value due to body bag emissions. A unique finding for body no. 7 is the detection of the fatty acids oleic acid, stearic acid, and palmitic acid, the latter determined in highest concentrations. The combination of these three compounds is commonly released during the splitting of fats [27] and might be addressed to specific ambient conditions like high temperatures during death. In addition, several alcohols and aldehydes are associated with the breakdown of lipids through oxidative reactions [33]. In addition, just body no. 7 released urea in low concentrations (14 ± 6 µg m^−3^). It can occur as a metabolic product or as a component of barbiturates (sleeping pills, narcotic agents). Moreover, the substance methyl thiocyanate was emitted by body no. 7 (139 ± 69 µg m^−3^). It is difficult to trace this substance back to a specific source. As it is used as insecticide agent and fumigant, it can be assumed to be part of the impregnation of the tent (see Table 1).

The findings confirmed that DMDS and DMTS are released in high concentrations at advanced decomposition, whereas DMDS levels were significantly higher by a factor of 2 to 4 than those of DMTS. However, the assumption that levels of sulfurous compounds will be higher at that stage due to advanced decomposition is not entirely valid since also in early stages of decay sulfurous substances were be detected in high concentration ranges, such as e.g. for body no. 4 (stage 2). In comparison to stages 1 and 2, nitrogenous substances were emitted by all bodies of stage 3 in significant levels (560 ± 107 µg m^−3^ to 1975 ± 57 µg m^−3^). This substance group can be traced back to the deamination of proteins [1, 27]. Trimethylamine and (dimethylamino)acetonitrile occurred as main abundant substances. Pyridine was detected in the air of five body bags (stages 1-3) and mostly in low or trace concentrations (1–22 µg m^−3^). It was just determined in higher levels in body bag air of body no. 4 (143 ± 2 µg m^−3^). Similar to the other stages, the same range of terpenes was identified with highest levels detected in body bag air of body no. 8 with alpha-pinene (1365 ± 45 µg m^−3^) and 3-carene (913 ± 57 µg m^−3^) as most abundant substances. Several short-branched alcohols are fermentation products of the amino acids valine, leucine and isoleucine and are formed via the Ehrlich pathway, such as 1-propanol, isobutanol, 2-methyl-1-butanol and 3-methyl-1-butanol [28, 33]. None of the bodies emitted all these compounds in parallel, but just few of them. Whereas low concentrations were measured in body bags of stages 1 and 2, higher levels were released at advanced decay with isobutanol and 3-methyl-1-butanol determined as main substances.

With the exception of body no. 7, aromatic hydrocarbons were released in much lower concentrations so the levels decreased from stage 1 to stage 3. Thus, it can be assumed that decomposition of aromatic amino acids starts right after dead and decreases with advanced decay. The process seems nearly to be finished at advanced decay. Indole was just released by bodies no. 6, 7 and 9 in low concentrations. It is known to be formed by the breakdown of phenylalanine, tryptophan and tyrosine. The same applies for skatole, which was not detected during the experiments [33].

### Olfactory signatures

**Fig. 5:**
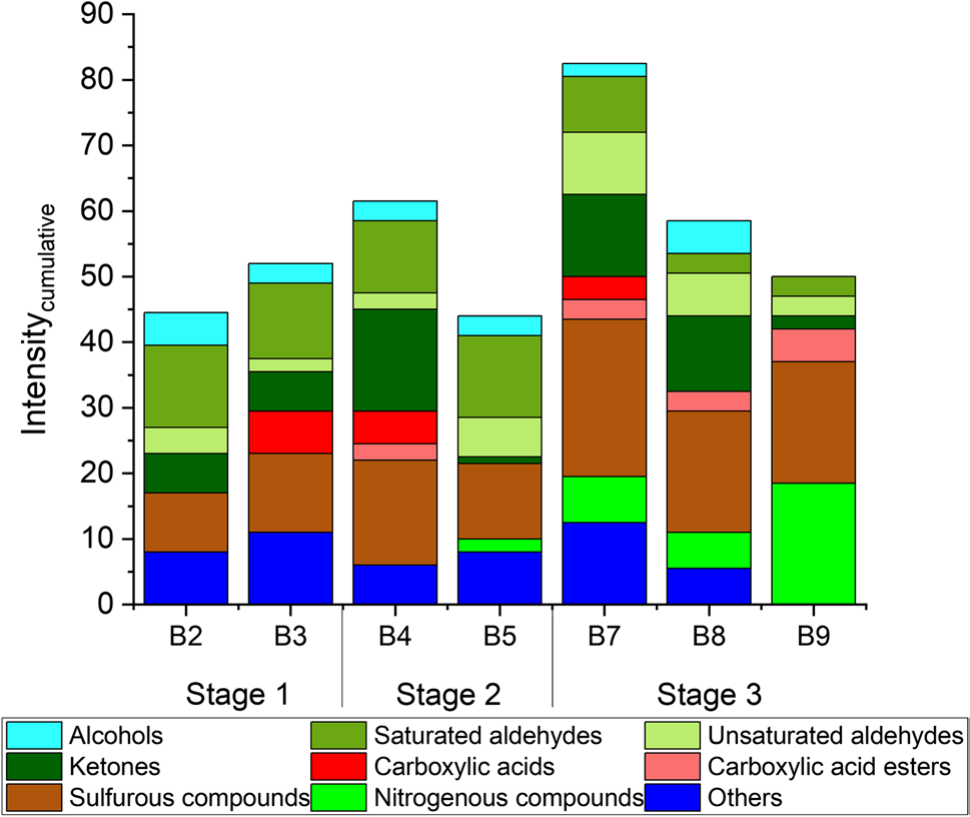
Cumulative intensity of odour active substances identified by GC-O after active sampling of body bag air. Bx: Body number x

The air in seven body bags was additionally sampled for GC-O analysis (see Table 1). Three samples belonged to decomposition stage 3 (advanced decomposition), two to stage 2 (initial decomposition) and another two to stage 1 (fresh bodies). Figure 5 visualizes the cumulative intensity of odour active substance groups identified by GC-O. As shown, the sulfurous compounds contributed most to the odour perception of the investigated air samples. A slight increase in intensity was observed from decomposition stage 1 to stage 3. The sulfurous substances dimethyl sulfide (DMS), dimethyl disulfide (DMDS) and dimethyl trisulfide (DMTS), which are well-known for their unpleasant sulfurous, cabbage-like odour, were identified in all samples and evaluated as significantly too strong (intensity stage 3 to 4), see Fig. 5. As an exception, for body no. 2 (stage 1) DMS was just detected in minor concentrations (20 ± 7 µg m^−3^) by sampling VVOCs on Carbograph™ 5TD. It was neither identified by GC-MS according to ISO 16000-6 [22] nor by GC-O after sampling on Tenax® TA. However, the fact that several sulfurous compounds were identified by GC-O for stage 1 bodies shows that even those substances, which were not identified as most abundant compounds, were clearly perceptible due to their very low odour threshold. In addition, carbon disulfide was detected in stage 2 (body no. 4) and stage 3 (bodies no. 7, 8 and 9) samples, whose odour patterns were described as rotten, cheesy and fishy. Moreover, another sulfurous substance was identified in all air samples at a retention time of ~ 20 min (RI ~ 1245). Based on the mass spectrum and the odour description, it can be assumed to be dimethyl tetrasulfide. However, a clear identification was not possible because no liquid analytical standard was available. The same applied for a sulfurous substance which was detected at a retention time of ~ 7 min (RI ~ 815) in air samples of bodies no. 8 and 9 (stage 3) which could be perhaps addressed to methyl ethyl disulfide. Body no. 7 (stage 3) also released methyl isocyanate, which was just detected by GC-O and which has a strong and unpleasant odour described as sharp and radish-like. 3-(Methylthio)propione as further strong smelly substance with a fatty, cheesy, bready and vegetable odour type was identified by GC-O as emissions from bodies no. 4 (stage 2) and no. 3 (stage 1).

Apart from the sulfurous substances, nitrogenous compounds were also identified. However, the nitrogenous substance trimethylamine was almost exclusively identified in decomposition stage 3. The exception was sample no. 5 (stage 2), where this strongly fishy smelly compound could also be detected. The sample air of body no. 9 (stage 3) contained, in addition to trimethylamine, the odour active compounds isobutylamine, (dimethylamino)acetonitrile, 2,5-dimethylpyrazine, 2-acetyl-1-pyrroline (only detectable via GC-O) as well as the substance pyrazine. All substances reveal an unpleasant odour quality and are perceived by the human nose as fishy, cheesy (trimethylamine, isobutylamine, (dimethylamino)acetonitrile) or toasty as well as roasted (2,5-dimethylpyrazine, 2-actyl-1-pyrroline, pyrazine). Also, the aromatic hydrocarbon phenol, which was identified in all stages of decomposition, was detected by GC-O due to its unpleasant, rubber- and plastic-like odour (intensity stage 3). An intensive unpleasant odour can be also caused by (un-)saturated aldehydes due to their low odour thresholds. The human nose has a higher detection sensitivity for these substances than the routine GC-MS method. Thus, several (un-)saturated aldehydes were just detected by GC-O and could only be partially identified by GC-MS, such as ethyl acrolein, trans-2-hexenal, octanal and nonanal. The substance groups of (un-)saturated aldehydes were associated with an unpleasant, strong, fatty and cheesy odour.

Ketones were also detected in trace concentrations by the human nose and caused a noteworthy contribution to the odour perception of bodies no. 7, 8 (stage 3) and body no. 4 (stage 2) with an intensity of about 2 to 3. However, ketones played a rather minor role in the odour perception of bodies no. 9 (stage 3) and no. 5 (stage 2). In nearly all air samples, 2-butanone was detected which is described as ethereal, fruity and camphor-like smelly substance. An exception was the air sample of body no. 5 (stage 2), in which 2-butanone could not be identified by GC-O. Moreover, in several samples further ketones were determined and confirmed via GC-MS, such as 2-pentanone, acetophenone, 3-methyl-2-butanone (MIPK) and 3-hydroxy-2-butanone. Substances with low odour thresholds are also the carboxylic acids butanoic acid and valeric acid. It is important to highlight that very polar carboxylic acids are relatively difficult to identify on a non-polar GC-MS column. Moreover, several intensive smelling alcohols have been detected, such as e.g. isobutanol, 1-butanol, 3-methyl-3-buten-1-ol. As shown in Fig. 5, this substance group has a minor odour impact of the seven examined body samples. Furthermore, a large number of unknown compounds was detected by GC-O and might be contributors for the overall odour of the samples. However, since these compounds were not detected by GC-MS analysis and because there was no indication from the internal odour database (retention index, odour type description), these compounds could not be identified further.

## Overall observations

As the concentration levels vary in a large range, also within the decomposition stages, the data illustrate the difficulty in identifying a unique “fingerprint” for the different decomposition stages of human bodies, as no sharp differentiation is possible. Also, no relation between the measured emission patterns and the causes and circumstances of death were identified. Even though the majority of detected compounds was similar for all stages, it is not easy to define “key substances” as done in other studies [2–4, 13, 37, 38]. Rosier et al. [2, 13] highlighted 5 organic esters as key compounds to separate human from pig remains. However, the experiments were performed on small samples located in glass jars of approx. 1 L volume. None of the substances listed as key substances were identified in this study, which underlines that experimental results obtained by using tissue samples cannot be transferred to the emissions of complete bodies. In addition, the samples were investigated over a long period (9 and 12 months) which is not a realistic scenario since a human body will decompose and degrade in a few weeks to months under natural, not controlled conditions. Just few of the core substances highlighted by Statheropoulos et al. [3, 4] were also identified in the present study. Here, DMDS and DMTS were evaluated as key compounds [3] and confirmed by the present data. DMDS occurred in significant concentrations already shortly after death and was emitted by all bodies of all stages in increased concentrations. Levels were by a factor of 2 to 20 higher than the values detected for DMTS. However, since organic sulfides can be released into indoor air by several environmental emission sources, such as e.g. cooked food, mould growth, bacterial contamination or human faeces [3, 39], the question remains open whether it will be possible to easily locate hidden human remains based on air analysis. It should also be noted that the location of the human body with its environmental parameters and weather conditions also plays an important role in the body’s emission behaviour. In the present study, a range of terpenes were detected in a characteristic pattern over all decomposition stages. It is very surprising that these substances were not identified in other studies, which highlights the novelty of this finding. As there are no published data, the specific metabolic processes leading to the formation and release of terpenes remains open. Nitrogenous substances can also be evaluated as a dominant substance group on the basis of the present findings. Interestingly, similar emissions were not reported in previous studies focussing on complete bodies [3]. By contrast, the biogenic amines putrescine and cadaverine were not detected. Both substances originate from the decarboxylation of amino acids, namely lysine and ornithine, and are assumed to be responsible for the typical “smell of death” [40]. However, it is well-known that both substances cannot be detected by classical GC-MS analysis, but no other reliable analytical technique has been published so far [1]. Cooke et al. [41] analysed cadaverine and putrescine in human saliva samples by high performance liquid chromatography (HPLC) after derivatization.

The present study can serve as a basis for substantial improvements in forensic science. HRD dogs need to be trained systematically, and one of the challenges is to provide a consistent quality of scent sources that are of realistic quality and composition [11, 42, 43]. Moreover, it provides a good overview of chemical substances that could be used systematically and in consistent quality for such a scientific “training cocktail”—and which at the same time could make it possible to dispense with real human tissue as far as possible due to ethical and legal restrictions. In addition to the detection of human corpses and remains, the determination of the time since death is another possible application of chemical profiles of different stages of decomposition [44]. Although intensive research has been conducted for more than a century to determine the post-mortem interval [45], in the earlier past with new approaches concerning changes in biochemical constituents in different body tissues or body fluids [46, 47], DNA/RNA degradation [48, 49] or the post-mortem microbiome [50], this topic remains challenging. So far, entomology is the only proven and accepted tool for estimating a minimum period in the late interval and there is the need for more methods [44, 51]. That the analysis of VOCs may in principle be useful to narrow down the PMI has already been shown by various authors. The majority of studies published so far focused on human surrogates like pigs or did not analyse real data due to experimental settings. However, Knobel et al. [20] clearly showed variations and dissimilarities in the composition and abundance of VOCs over time between the odour profiles of pig cadavers and human bodies, showing the need for human specific decomposition and VOC data sets. In contrast, Stefanuto et al. [52] and Perrault et al. [38] found different VOC compositions between gas samples obtained minimally invasively from body cavities of four, respectively five different human bodies and attributed this either to the circumstances of death and/or to the PMI and called for further research in this regard. In this context, the present study contributes for the first time human-specific field data on a larger scale and of different post-mortem intervals.

## Conclusions and outlook

This study presents comprehensive emission measurements of deceased human bodies in different decomposition stages and at controlled environmental conditions. The obtained results show that decaying human bodies are very strong emission sources by releasing a great variety of organic volatiles, even shortly after death. Several substances were identified, which were already reported in previous studies, notably sulfurous and nitrogenous substances as well as branched (cyclo-)alkanes, aldehydes, ketones, alcohols, carboxylic acids, carboxylic acid esters and ethers. In addition, several terpenes were identified which were released in a similar pattern mainly at advanced decomposition (stages 2 and 3). This is a new finding as no comparable data have been published before. Facing the variety of detected volatile organics, it becomes clear how complicated the definition of key substances for different decomposition stages is. Additional experiments with a larger number of bodies under defined laboratory conditions are necessary in order to differentiate characteristic substances from those of minor importance. Based on this, further investigations targeting on varying environmental parameters should be performed stepwise, considering e.g. different indoor environments as well as outdoor and weathering conditions. Moreover, individual characteristics such as diseases, nutritional habits and clothing need to be considered. It was also shown that it is of high importance to perform emission testing of enclosures in which the test specimens are located in order to differentiate background pollutants from released substances. The findings demonstrate that using body bags as a kind of “emission test chamber” is a very promising approach since the body bag materials are diffusion-tight and preventing contamination from room air. Moreover, it is a real application when it comes to transport and store of a body before autopsy. However, at the same time it is limited by the fact that undertakers use different kind of body bags, which requires emission testing of each body bag material.

Furthermore, all investigated samples were very odour-intensive sources with a large number of different odour active components in a partly high concentration range. Consequently, the identification of individual odour active compounds in very low concentration ranges was difficult to realize. Another difficulty is the identification of odour active substances due to olfactory adaptation in samples with a large number of VOCs. However, GC-O analysis shows that the chemical groups, such as the sulfurous compounds, unsaturated aldehydes and the ketones, have a great influence on the overall odour of the human corpse in all three different stages of decomposition. This contrasts with the (V)VOCs detected by GC–MS. Here, the main components of the examined bodies were mostly alcohols, but they play a subordinate role in the GC-O analysis. Therefore, the focus for the development of new methods for detecting deceased odours should be targeted on the strong-smelling components mentioned above. Ideally, target substances are identified that are already perceived in trace concentrations by the human nose through a specific odour type and can be detected unproblematically by standard analytical methods.

## Data Availability

Data will be made available on request.
